# Increased Nasopharyngeal Density and Concurrent Carriage of *Streptococcus pneumoniae*, *Haemophilus influenzae*, and *Moraxella catarrhalis* Are Associated with Pneumonia in Febrile Children

**DOI:** 10.1371/journal.pone.0167725

**Published:** 2016-12-01

**Authors:** Sopio Chochua, Valérie D'Acremont, Christiane Hanke, David Alfa, Joshua Shak, Mary Kilowoko, Esther Kyungu, Laurent Kaiser, Blaise Genton, Keith P. Klugman, Jorge E. Vidal

**Affiliations:** 1 Hubert Department of Global Health, Rollins School of Public Health, Emory University, Atlanta, Georgia, United States of America; 2 Swiss Tropical and Public Health Institute and University of Basel, Basel, Switzerland; 3 Department of Ambulatory Care and Community Medicine, University of Lausanne, Lausanne, Switzerland; 4 Amana Hospital, Dar es Salaam, United Republic of Tanzania; 5 St. Francis Hospital, Ifakara, United Republic of Tanzania; 6 Laboratory of Virology, Division of Infectious Diseases and Division of Laboratory Medicine, University Hospital of Geneva, and Faculty of Medicine, University of Geneva, Geneva, Switzerland; 7 Infectious Diseases Service, University Hospital, Lausanne, Switzerland; University of Malaya, MALAYSIA

## Abstract

**Background:**

We assessed nasopharyngeal (NP) carriage of five pathogens in febrile children with and without acute respiratory infection (ARI) of the upper (URTI) or lower tract, attending health facilities in Tanzania.

**Methods:**

NP swabs collected from children (N = 960) aged 2 months to 10 years, and with a temperature ≥38°C, were utilized to quantify bacterial density of *S*. *pneumoniae (Sp)*, *H*. *influenzae (Hi)*, *M*. *catarrhalis (Mc)*, *S*. *aureus (Sa)*, *and N*. *meningitidis (Nm)*. We determined associations between presence of individual species, densities, or concurrent carriage of all species combination with respiratory diseases including clinical pneumonia, pneumonia with normal chest radiography (CXR) and endpoint pneumonia.

**Results:**

Individual carriage, and NP density, of *Sp*, *Hi*, or *Mc*, but not *Sa*, *or Nm*, was significantly associated with febrile ARI and clinical pneumonia when compared to febrile non-ARI episodes. Density was also significantly increased in severe pneumonia when compared to mild URTI (*Sp*, *p*<0.002; *Hi p*<0.001; *Mc*, *p* = 0.014). Accordingly, concurrent carriage of *Sp*^*+*^, *Hi*^*+*^, and *Mc*^*+*^, in the absence of *Sa*^*-*^ and *Nm*^*-*^, was significantly more prevalent in children with ARI (*p* = 0.03), or clinical pneumonia (*p*<0.001) than non-ARI, and in children with clinical pneumonia (*p* = 0.0007) than URTI. Furthermore, *Sp*^*+*^, *Hi*^*+*^, and *Mc*^*+*^ differentiated children with pneumonia with normal CXR, or endpoint pneumonia, from those with URTI, and non-ARI cases.

**Conclusions:**

Concurrent NP carriage of *Sp*, *Hi*, and *Mc* was a predictor of clinical pneumonia and identified children with pneumonia with normal CXR and endpoint pneumonia from those with febrile URTI, or non-ARI episodes.

## Introduction

The nasopharynx is an ecologic reservoir for human bacterial pathogens such as *Streptococcus pneumoniae* (*Sp*), *Moraxella catarrhalis* (*Mc*), *Haemophilus influenzae* (*Hi*), *Staphylococcus aureus* (*Sa*), and *Neisseria meningitidis* (*Nm*) [1]. Whereas these species form part of the nasopharyngeal microbiome [1, 2], they are also the source of several of the most prevalent causes of morbidity and mortality to human kind, which include diseases such as acute otitis media, pneumonia, bacteremia and meningitis [3].

Carriage of these nasopharyngeal species in healthy children varies amongst different studies and geographic regions [4]. In general, carriage prevalence of these bacteria is lower in industrialized countries than in resource-limited nations. The tendency, however, is that carriage of *Sp*, *Hi* or *Mc* increases during childhood, peaking at the age of 3 years, and then decreases [1]. Conversely, *Nm* carriage is low during childhood, but peaks in prevalence in young adults [5], whereas nasopharyngeal carriage of *Sa* decreases during childhood and remains relatively low thereafter [6, 7].

There are limited studies focused on investigating nasopharyngeal carriage during disease episodes. Studies conducted in Vietnamese children (<2 years old) showed a similar prevalence of nasopharyngeal carriage of *Sp*, *Hi* or *Mc* in children with pneumonia compared to healthy controls but an increased *Sp* nasopharyngeal density was observed in pneumonia patients, compared to controls [8]. Carriage of *Sp*, *Hi* or *Mc* has also been associated with the development of otitis media and sinusitis [9, 10]. An increased nasopharyngeal carriage of *Sp* has also been associated with infection with influenza virus, rhinovirus, and adenovirus in admitted South African children with pneumonia [11] and to influenza virus and parainfluenza virus in Peruvian children with acute respiratory infection (ARI) [12].

The complex milieu of these nasopharyngeal (NP) pathogens can also be modified by factors such as the use of antimicrobial medicines or vaccines, or the innate immune response, which include the development of an acute infection [13, 14]. To the best of our knowledge, carriage dynamics by all these five species (i.e., *Sp*, *Mc*, *Hi*, *Sa* and *Nm*) have not been previously investigated at the same time in the nasopharynx of ill children. The present study investigated carriage of these five major human pathogens in a cohort of urban and rural Tanzanian children presenting with an acute febrile illness [15]. The associations between presence of these bacteria, concurrent carriage or nasopharyngeal densities, and disease conditions were assessed. More precisely, we investigated differences in bacterial carriage according to: 1) respiratory disease versus other type of infections causing fever, 2) clinical pneumonia versus upper respiratory tract infections, 3) type of radiological findings in children with clinical pneumonia, and 4) disease severity.

## Material and Methods

### Study area and population

Nasopharyngeal swabs (NP) were collected from 1005 febrile children, aged 2 months to 10 years, presenting at the outpatient clinic of Amana District Hospital in the economical capital of Tanzania, Dar es Salaam, and at the outpatient clinic of St. Francis Designated District Hospital in Ifakara, Kilombero District, a small rural town in South central Tanzania, as described elsewhere [15]. Briefly, the enrollment period was from April to August 2008 for patients of Dar es Salaam and from June to December 2008 for those of Ifakara. Children with an axillary temperature of ≥ 38°C and requiring no immediate lifesaving procedures were assessed for inclusion criteria: 1) first visit for the present illness, 2) fever duration ≤ 1 week, 3) chief reason for visit not injury/trauma, 4) no antimalarial or antibiotic received during the preceding week, and 5) no severe malnutrition. A questionnaire and clinical examination were administered. At the time of study, enrollment in the Expanded Program on Immunization in Tanzania did not include the pneumococcal vaccine or *Haemophilus influenzae* vaccine. Ethical approval by the Institutional Review Board of the Ifakara Health Institute (IHI/IRB/No. A 60) and the National Institute for Medical Research Review Board (NIMR/HQ/R.8a/Vol.IX/823) in Tanzania, as well as the Ethikkommission beider Basel (EKBB 130/09) in Switzerland.

### Definition of febrile diseases

Final diagnosis(ses) for acute febrile illness was established based on criteria from the World Health Organization (WHO), Infectious Diseases Society of America guidelines and systematic reviews [15]. Acute respiratory infection (ARI) was defined as any acute (≤1 week) infection manifested by at least one respiratory sign or symptom localized to the upper or lower respiratory tract and divided in two categories: clinical pneumonia or upper respiratory tract infection (URTI). Children with clinical pneumonia were further divided in three categories based on chest radiography (CXR) findings, according to the WHO Pneumococcal Trials Ad Hoc Committee recommendations [16, 17]: alveolar consolidation and/or pleural effusion was categorized as endpoint pneumonia; other infiltrates (that in all these children corresponded to peribronchial thickening +/- atelectasis, compatible with the clinical entity of bronchiolitis), were categorized as pneumonia with other infiltrates; normal radiography was categorized as pneumonia with normal CXR. Severe febrile disease (whenever due to ARI or another type of infection) was defined as the presence of at least one of the following features: respiratory distress, impaired consciousness, seizures, meningismus, cardiovascular failure, renal failure, severe anemia (hemoglobin <5 g/dl), severe dehydration, jaundice, and severe malnutrition.

### Specimen collection and storage

NP swabs were collected according to recommendations from the WHO [18] and immediately stored in 1 ml of STGG (skim-milk, tryptone, glucose and glycerol) transport medium [19], vortexed for 20 s with the swabs inside to release bacteria into the STGG and frozen at -80°C until further analysis.

### DNA extraction from nasopharyngeal swabs

All DNA extractions were performed in an access-restricted laboratory room utilized only for processing clinical samples and under a biological safety cabinet with sterile environment. Frozen NP samples were thawed at room temperature and then vortexed for 15 s. Two hundred microliters of the sample were mixed with 100 μl of TE buffer (10mM Tris-HCl, 1 mM EDTA, pH 8.0) containing 0.04 g/ml of lysozyme and 75 U/ml of mutanolysin, and then incubated for 1 h in a 37°C in water bath. The subsequent steps were carried out according to the Qiagen DNA mini kit protocol, as detailed elsewhere [20, 21]. DNAs were eluted in 100 μl of elution buffer and stored at -80°C. DNA from reference strains *Sp* (TIGR4), *Sa* (American Type Culture Collection (ATCC) 25923), *Hi* (Centers for Disease Control and Prevention (CDC) reference strain M5216), *Mc* (CDC reference strain M15757), and *Nm* (CDC8201085) [22] were also extracted from overnight cultures using the QIAamp kit. DNA concentration was measured by the Nanodrop method (Nanodrop Technologies, Wilmington, DE).

### Quantitative PCR

Quantitative PCR (qPCR) assays targeting the following genes *lyt*A, *nuc*, *hpd*, *cop*B, and *sod*C, carried by all *Sp*, *Sa*, *Hi*, *Mc*, and *Nm* respectively, were performed. The qPCR assays utilized published primers and probes at concentrations optimized in this study and shown in Table 1 [23–28].

**Table 1:** Primers and probes utilized in this study.

Target Gene		Sequence	Probe fluorophore and quencher	Concentration (nM)	Reference
*lytA*	Forward	ACGCAATCTAGCAGATGAAGCA		200	[23]
	Reverse	TCGTGCGTTTTAATTCCAGCT		200	
	Probe	TGCCGAAAACGCTTGATACAGGGAG	5' FAM, 3' BHQ1	200	
*nuc*	Forward	GTTGCTTAGTGTTAACTTTAGTTGTA		400	[26]
	Reverse	AATGTCGCAGGTTCTTTATGTAATTT		400	
	Probe	FAM-AAGTCTAAGTAGCTCAGCAAATGCA	5' HEX, 3' BHQ1	200	
*sodC*	Forward	GCACACTTAGGTGATTTACCTGCAT		400	35
	Reverse	CCACCCGTGTGGATCATAATAGA		400	
	Probe	CATGATGGCACAGCAACAAATCCTGTTT	5' TEXAS RED, 3' BHQ1	200	
*hpd*	Forward	GGTTAAATATGCCGATGGTGTTG		300	[27]
	Reverse	TGCATCTTTACGCACGGTGTA		300	
	Probe	TTGTGTACACTCCGT“T”GGTAAAAGAACTTGCAC	5' FAM, BHQ1dT, 3' SPC6	100	
*copB*	Forward	CGTGTTGACCGTTTTGACTTT		200	[25]
	Reverse	TAGATTAGGTTACCGCTGACG		200	
	Probe	ACCGACATCAACCCAAGCTTTGG	5' HEX, 3' BHQ1	100	

Quantitative PCR reactions were carried out in a final 25 μl volume and performed using Platinum qPCR superMix (Invitrogen), according to the instructions of the manufacturer, with 2.5 μl of purified DNA and the concentration of each primer and probe set shown in Table 1. A no-template control was always included in every run. To quantify the number of genome copies present in each sample, purified genomic DNA from the corresponding reference strain was serially diluted with TE and run in parallel. Genome copies of each set of standards were as follows: *Sp* (TIGR4) 2.14, 2.14x10^1^, 4.29x10^1^, 4.29x10^2^, 4.29x10^3^, 4.29x10^4^, 4.29x10^5^; *Sa* (ATCC 25923) 1.64, 1.64x10^1^, 3.29x10^1^, 3.29x10^2^, 3.29x10^3^, 3.29x10^4^, 3.29x10^5^; *Hi (*CDC M5216) 2.53, 2.53x10^1^, 5.06x10^1^, 5.06x10^2^, 5.06x10^3^, 5.06x10^4^, 5.06x10^5^; *Mc* (CDC M15757) 2.49, 2.49x10^1^, 4.97x10^1^, 4.97x10^2^, 4.97x10^3^, 4.97x10^4^, 4.97x10^5^, and *Nm* (CDC8201085) 2.14, 2.14x10^1^, 4.29x10^1^, 4.29x10^2^, 4.29x10^3^, 4.29x10^4^, 4.29x10^5^. These standards were run along with DNA from NP samples in a CFX96 real time system (BioRad, Hercules, CA) and CFU/ml were calculated using the software Bio-Rad CFX manager. The following cycling parameters were utilized: 95°C for 2 min, followed by 40 cycles of 95°C for 15 s and 60°C for 1 min. Negative samples were defined with cycle threshold (CT) values, if any, greater than >40. All reactions showed an efficiency between 94 and 98% (recommended 90–110%) [29].

### Statistical analysis

The main outcomes of interest were the relationships of carriage with the presence of a certain febrile disease. Chi-square tests were performed to examine whether there was an association among bacterial densities of all bacteria, and between each bacteria, and other independent categorical variables in the dataset. Logistic regression models were used to determine if carriage of one bacterial species was associated with carriage of the other species. To examine the effects of covariates on each species, we modeled carriage of all five bacteria separately. Carriage of *Sp*, *Hi*, *Sa*, *Mc*, or *Nm* was modeled separately, and each model included the presence/absence of the other species as the main exposures of interest and adjusted for age, sex, site, and ARI. Evaluation of models was done using the ‘Goodness of fit’ tests such as the Hosmer-Lemeshow test. All statistical analyses were performed using SAS version 9.4 (SAS Institute, Inc., Cary, NC, USA) or SigmaPlot for Windows Version 12.0 (Systat Software, Inc.).

## Results

### Demographic and clinical characteristics of the febrile children

A total of 1005 febrile children were consecutively recruited. Nine hundred and sixty febrile children, for whom enough NP material was available, were included in this study. Demographic and clinical characteristics of these children are described in Table 2. Among the children included in the analysis, 62% presented at the clinic with an ARI (44% with URTI, 12% with pneumonia with normal CXR, 3% with pneumonia with infiltrates and 3% with endpoint pneumonia), whereas 23% were diagnosed with other diseases, and 15% had unknown disease. About 6% of children had malaria, 6% gastrointestinal disease, 2% typhoid, 4% urinary tract infection, 5% systemic disease and less than 1% presented with occult bacteremia, or skin disease. Overall, 13% of the children had a severe febrile illness, and among children with ARI, 10.6% had a severe presentation (Table 2).

**Table 2:** Demographic and Clinical Characteristics of Febrile Children (N = 960).

Characteristic	Number (%)
Sex	
Male	491 (51.1)
Female	469 (48.9)
Age (month)	
≤ 12	312 (32.5)
> 12–36	432 (45.0)
> 36	216 (22.5)
Site	
Rural (Ifakara)	481 (50.1)
Urban (Dar es Salaam)	479 (49.9)
Acute respiratory infection	597 (62.2)
Upper respiratory tract infection	420 (43.8)
Clinical pneumonia	177 (18.4)
Pneumonia with normal CXR	117 (12.2)
Pneumonia with other infiltrates	33 (3.4)
End-point pneumonia	27 (2.8)
Other disease	220 (22.9)*
Malaria	60 (6.3)
Gastrointestinal infection	57 (5.9)
Typhoid	19 (2.0)
Urinary tract infection	39 (4.1)
Skin infection	9 (0.9)
Occult bacteremia^#^	7 (0.7)
Other systemic infection	51 (5.3)
Unknown disease	143 (14.9)
Severe disease	124 (12.9)

* Note: total of the other disease sum (220) does not equal to the sum of individual diseases (242) summed up, because some children have more than one category of the other disease;

^#^defined as a child with a positive blood culture other than *Salmonella typhi*, without a localized source of infection

### Prevalence of individual nasopharyngeal carriage of bacterial pathogens and association with respiratory infections

The overall prevalence of nasopharyngeal carriage of *Sp* was 81%, *Hi* 75%, *Sa* 23%, *Mc* 91%, and *Nm* 51% (Table 3). However, when detection of these species was individually assessed in each disease category, *Sp*, *Hi* or *Mc* was more prevalent in the nasopharynx in cases of febrile ARI, in comparison to febrile non-ARI cases [*Sp* (odds ratio, OR = 1.76, 95% confidence interval, CI: 1.27–2.44), *Hi* (1.91, 1.42–2.56) or *Mc* (1.65, 1.06–2.56)], as well as in cases of clinical pneumonia when compared to non-ARI cases [*Sp* (2.32, 1.40–3.85), *Hi* (2.55, 1.62–4.01) or *Mc* (3.26, 1.44–7.41)] (Table 3). Carriage rates of individual species when comparing the different types of respiratory disease, or disease severity, for example URTI vs clinical pneumonia, end-point vs other infiltrates, end-point pneumonia vs normal CXR pneumonia, was similar (data not shown).

**Table 3:** Prevalence of nasopharyngeal carriage by bacterial species and their association with respiratory infections.

Bacterial pathogen	Overall (N = 960)	Non-ARI (N = 363)	ARI (N = 597)	Odds Ratio* (95% CI) P-value (2-tail)	Clinical pneumonia (N = 177)	Odds Ratio** (95% CI) P-value (2-tail)
*S*. *pneumoniae*	776 (80.8%)	273 (75.2%)	503 (84.3%)	1.76 (1.27–2.44) 6.5x10^-4^	155 (87.5%)	2.32 (1.40–3.85) 6.5x10^-4^
*H*. *influenzae*	715 (74.5%)	242 (66.7%)	473 (79.2%)	1.91 (1.42–2.56) 1.8x10^-5^	148 (83.6%)	2.55 (1.62–4.01) 2.3x10^-5^
*S*. *aureus*	223 (23.2%)	95 (26.2%)	128 (21.4%)	0.77 (0.57–1.05) 9.4x10^-2^	35 (19.8%)	0.69 (0.44–1.07) 1.0x10^-1^0.1021
*M*. *catarrhalis*	872 (90.8%)	320 (88.1%)	552 (92.5%)	1.65 (1.06–2.56) 2.7x10^-2^	170 (96.0%)	3.26 (1.44–7.41) 1.9x10^-3^
*N*. *meningitidis*	492 (51.2%)	190 (52.3%)	302 (50.6%)	0.93 (0.72–1.21) 5.9x10^-1^	82 (46.3%)	0.78 (0.54–1.12) 1.9x10^-1^

*Non-ARI vs ARI,

**Non-ARI vs clinical pneumonia

### Concurrent carriage of bacterial pathogens and association with respiratory infections

Further analyses revealed that 94.2% of febrile children enrolled were carrying more than one species of the five bacterial species tested in this study (Table 4). Concurrent carriage of *Sp*, *Hi*, and *Mc* was associated with respiratory infections (S1 Table). Carriage of all three *Sp*, *Hi*, and *Mc* [in the absence of *Sa* and *Nm* (*Sa*^-^ and *Nm*^-^)] was significantly more prevalent in the nasopharynx of children with febrile ARI than in children with a febrile non-ARI episode (p = 0.035) or in children with clinical pneumonia vs URTI (p = 0.009) (S1 Table). Furthermore, concurrent carriage of these three species, in the absence of *Sa*^-^ and *Nm*^-^, was also significantly more prevalent in the nasopharynx of children with pneumonia with normal CXR than those children with URTI (p = 0.018) (S1 Table). The sensitivity and specificity to differentiate pneumonia with normal CXR and endpoint pneumonia from non-ARI cases was 75% and 80% or 81% and 80%, respectively, whereas the sensitivity to differentiate pneumonia with normal CXR, or endpoint pneumonia, from URTI cases was 75% and 77%, or 81.5%, and 77.6%, respectively.

**Table 4:** Prevalence of concurrent carriage by bacterial species in the nasopharynx of febrile children.

Pattern	Bacterial species and combinations	Number (%)
	**No bacterial species identified**	24 (2.5)
	**One bacterial species identified**	56 (5.8)
Most prevalent	*M*. *catarrhalis*	26 (2.7)
Least prevalent	*H*. *influenzae*	6 (0.6)
	**Two bacterial species identified**	128 (13.3)
Most prevalent	*S*. *pneumoniae* + *M*. *catarrhalis*	53 (5.5)
Least prevalent	S. *pneumoniae* + *S*. *aureus*,*H*. *influenzae* + *S*. *aureus*	2 (0.2) 2 (0.2)
	**Three bacterial species identified**	325 (33.9)
Most prevalent	*S*. *pneumoniae* + *H*. *influenzae* + *M*. *catarrhalis*	223 (23.2)
Least prevalent	*H*. *influenzae + S*. *aureus* + *N*. *meningitidis*	1 (0.1)
	**Four bacterial species identified**	344 (35.8)
Most prevalent	*S*. *pneumoniae* + *H*. *influenzae* + *M*. *catarrhalis* +*N*. *meningitidis*	262 (27.3)
Least prevalent	*H*. *influenzae* + *M*. *catarrhalis* + *S*. *aureus* +*N*. *meningitidis*	15 (1.6)
	**All five bacterial species identified**	83 (8.6)

### Assessing the associations of nasopharyngeal carriage of *Sp*, *Hi*, *Sa*, *Mc*, *and Nm*

Logistic regression models of carriage (presence or absence) of *Sp*, *Hi*, *Mc*, *Nm*, *and Sa* are shown in Table 5. All models were controlled for age, gender, enrollment site and ARI. The model of carriage of *Sp* indicated a significant positive association between *Sp* and *Hi*, *Sp* and *Mc*, and *Sp* and *Nm*. The presence of *Sa* and age >36 months was negatively associated with carriage of *Sp*.

**Table 5:** Odds ratios with 95% confidence intervals for the associations between different bacterial species in the nasopharynx (adjusted for age, sex, site and ARI).

	*S*. *pneumoniae*	*H*. *influenzae*	*S*. *aureus*	*M*. *catarrhalis*	*N*. *meningitidis*
*S*. *pneumoniae*					
Absence (ref)	-	**2.46 (1.66–3.64)**	**0.53 (0.35–0.82)**	**5.22 (3.05–8.95)**	**1.54 (1.04–2.28)**
*H*. *influenzae*					
Absence (ref)	**2.41 (1.63–3.57)**	-	**0.66 (0.46–0.96)**	**3.34 (1.94–5.74)**	**1.55 (1.10–2.17)**
*S*. *aureus*					
Absence (ref)	**0.54 (0.35–0.83)**	**0.66 (0.45–0.96)**	-	1.36 (0.73–2.53)	**1.73 (1.25–2.40)**
*M*. *catarrhalis*					
Absence (ref)	**5.41 (3.18–9.21)**	**3.40 (2.00–5.78)**	1.40 (0.76–2.58)	-	1.66 (0.97–2.84)
*N*. *meningitidis*					
Absence (ref)	**1.49 (1.01–2.21)**	**1.56 (1.11–2.18)**	**1.73 (1.25–2.39)**	1.63 (0.95–2.83)	-
Sex					
Male (ref)	0.82 (0.57–1.19)	0.85 (0.61–1.17)	0.82 (0.60–1.11)	1.04 (0.63–1.74)	0.93 (0.71–1.21)
Site					
Rural (ref)	**0.29 (0.18–0.45)**	**0.42 (0.30–0.61)**	**0.59 (0.42–0.84)**	**0.11 (0.04–0.28)**	1.20 (0.88–1.62)
Age					
0–12 months (ref)					
>12–36 months	1.19 (0.75–1.89)	1.18 (0.80–1.74)	0.97 (0.67–1.40)	1.24 (0.66–2.32)	**3.70 (2.70–5.07)**
>36 months	**0.58 (0.36–0.94)**	0.89 (0.58–1.38)	1.04 (0.67–1.59)	1.24 (0.65–2.37)	**3.14 (2.16–4.56)**
Acute respiratory infection					
Absence (ref)	1.14 (0.78–1.66)	**1.43 (1.03–1.99)**	0.75 (0.54–1.03)	0.95 (0.56–1.59)	0.94 (0.71–1.26)

Note: significant associations are shown in bold

The model of *Hi* carriage showed a positive association between *Hi* and *Mc*, and *Hi* and *Nm*, while the presence of *Sa* was negatively associated with carriage of *Hi*. In the logistic regression model only the presence of *Hi* was associated with ARI. The model of *Sa* carriage showed that the presence of *Nm* was positively associated with carriage of *Sa*. There was no significant association found between carriage of *Sa* and *Mc*. The model of *Mc* carriage additionally showed no significant associations with the presence of *Nm*. Furthermore, the model of *Nm* carriage showed positive associations between *Nm* carriage and age > 12–36 months and > 36 months.

The odds of carrying *Sp*, *Hi*, *Sa*, *and Mc* in a child enrolled in the urban clinic was significantly less than that of a child enrolled in the rural clinic. This association was found regardless of the local prevalence of ARI among all febrile episodes (that was lower in the urban than in the rural site). All other possible covariates were assessed but found not to be significantly associated with bacterial carriage.

### Association between bacterial density and respiratory infections

**Fig 1:**
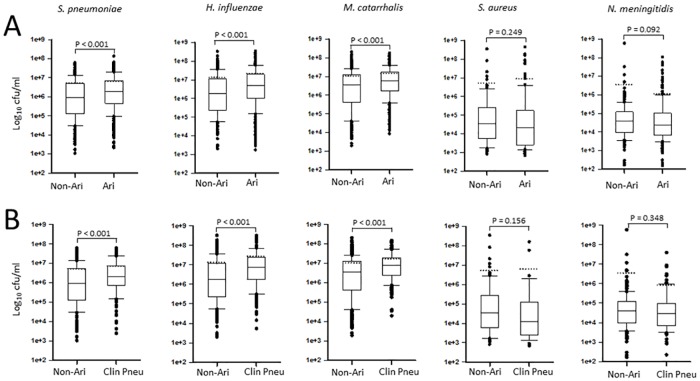
Nasopharyngeal density during non-ARI, ARI or clinical pneumonia in febrile Tanzanian children. The species analyzed is shown above each graphic. (A) ARI cases were compared against non-ARI. (B) Non-ARI cases were compared against children with clinical pneumonia (Clin Pneu). Statistical analyses were performed using the Mann-Whitney U test and showed significance for *S*. *pneumoniae*, *H*. *influenzae*, and *M*. *catarrhalis* in A and B. Dotted lines represent the means.

**Fig 2:**
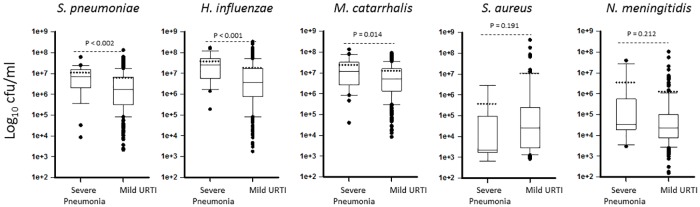
Nasopharyngeal density during severe pneumonia and mild URTI in febrile Tanzanian children. Nasopharyngeal density (cfu/ml) of each species in children with severe pneumonia were compared against children with mild upper respiratory tract infection (URTI). Statistical analyses were performed using the Mann-Whitney U test and showed significance for *S*. *pneumoniae*, *H*. *influenzae*, and *M*. *catarrhalis*. Dotted lines are the means.

Density was categorized according to increasing bacterial load. Table 6 shows that 64.0%, 75.1%, or 81.3% of children carried in the nasopharynx >1x10^6^ cfu/ml of *Sp*, *Hi* or *Mc*, respectively, whereas only 9.9%, or 13.3%, of children carried >1x10^6^ cfu/ml of *Nm* or *Sa*, respectively. We next investigated differences in nasopharyngeal density in children with respiratory infection in comparison to children with a non-ARI episode. As shown in Fig 1 and S2 Table, NP density of *Sp*, *Hi*, or *Mc*, was significantly higher in febrile ARI cases (clinical pneumonia or children with pneumonia with normal CXR) than in febrile non-ARI episodes [*p*<0.001 for all cases (S2 Table)]. Similarly, nasopharyngeal density of *Hi* or *Mc* was found significantly increased in clinical pneumonia when compared to URTI, (not shown). Nasopharyngeal density of *Sp*, *Hi* or *Mc*, was significantly higher in children with severe clinical pneumonia when compared to mild URTI (p<0.002, p<0.001 and p = 0.014 respectively) (Fig 2). Nasopharyngeal density of *Sa* and *Nm* were similarly detected in children with respiratory and non-respiratory infection.

**Table 6:** Prevalence of carriage by bacterial density overall and during ARI.

Bacterial density CFU/ml*	*S*. *pneumoniae*		*H*. *influenzae*		*S*. *aureus*		*M*. *catarrhalis*		*N*. *meningitidis*	
	Overall N (%)	ARI N (%)	Overall N (%)	ARI N (%)	Overall N (%)	ARI N (%)	Overall N (%)	ARI N (%)	Overall N (%)	ARI N (%)
0	184	94	245	124	737	469	88	45	468	295
≥10^2^–<10^3^	-	-	-	-	6 (2.7)	5 (3.9)	-	-	11 (2.2)	8 (2.6)
≥10^3^–<10^4^	30 (3.9)	12 (2.4)	13 (1.8)	8 (1.7)	75 (33.6)	45 (35.2)	11 (1.3)	1 (0.2)	136 (27.6)	91 (30.1)
≥10^4^–<10^5^	86 (11.1)	42 (8.3)	69 (9.7)	33 (7.0)	69 (30.9)	39 (30.5)	61 (7.0)	19 (3.4)	214 (43.5)	127 (42.1)
≥10^5^–<10^6^	202 (26.0)	127 (25.2)	134 (18.7)	77 (16.3)	42 (18.8)	22 (17.2)	135 (15.5)	83 (15.0)	90 (18.3)	46 (15.2)
≥10^6^–<10^7^	317 (40.9)	225 (44.7)	258 (36.1)	180 (38.1)	16 (7.2)	6 (4.7)	358 (41.1)	237 (42.9)	33 (6.7)	25 (8.3)
≥10^7^–<10^8^	140 (18.0)	96 (19.1)	212 (29.7)	151 (31.9)	11 (4.9)	8 (6.3)	298 (34.2)	206 (37.3)	6 (1.2)	4 (1.3)
≥10^8^–<10^9^	1 (0.1)	1 (0.2)	29 (4.1)	24 (5.1)	4 (1.8)	3 (2.3)	9 (1.0)	6 (1.1)	2 (0.4)	1 (0.3)

*Lowest limit of detection is 80 CFU/ml.

## Discussion

We have studied, for the first time, nasopharyngeal carriage of five major human bacterial pathogens, *Sp*, *Hi*, *Mc*, *Sa*, and *Nm* in children presenting the most common childhood syndrome: acute fever. Our analyses demonstrate that Tanzanian febrile children experiencing an episode of any type of ARI (including URTI, clinical pneumonia or pneumonia with normal CXR) carried significantly more *Sp*, *Hi*, *and Mc*, at the same time in the nasopharynx, in the absence of *Sa* and *Nm* than children with non-ARI infections. Accordingly, bacterial densities of these three species were found to be significantly increased in the nasopharynx of children with respiratory infection, when compared to those with non-ARI infections. Such a significantly higher prevalence, or increased density, was not observed in children carrying *Sa* or *Nm*.

Carriage studies including bacterial species investigated here, in children with ARI, have increased the last few years. The high prevalence of nasopharyngeal carriage of *Sp*, *Hi*, and *Mc* (87.5%, 82.6%, and 96.5%, respectively) in children with pneumonia, whether clinical or radiological, that we observed, differs from that obtained in a recent study of Vietnamese children with radiological pneumonia, whose carriage prevalence was 38.7, 50 and 28.1% for *Sp*, *Hi* and *Mc*, respectively [8]. A study by Wolter *et al* (2014) analyzed South African children experiencing invasive pneumococcal pneumonia and showed a 53% prevalence of nasopharyngeal carriage of the pneumococcus [11].

An increased nasopharyngeal pneumococcal density has been associated with pneumococcal pneumonia in HIV-infected adults [30] and children with pneumococcal or radiological pneumonia [8, 11, 30]. In line with this evidence, our study demonstrates a statistically higher pneumococcal density in the nasopharynx of children with ARI (median, 2.25x10^6^ cfu/ml), URTI (median, 1.73x10^6^ cfu/ml), clinical pneumonia (median, 2.05x10^6^ cfu/ml), and pneumonia with normal CXR (median, 1.90x10^6^ cfu/ml), when compared to those suffering from a non-ARI episode (median, 9.17x10^5^ cfu/ml), which represents a ∼2.5, ∼1.8, ∼2.2, or ∼2 fold-increase in pneumococcal density, respectively. Furthermore, we found a 4.2-fold increase of pneumococcal nasopharyngeal density when children with severe clinical pneumonia were compared to those with mild URTI, and a statistically significant high density in four children who died of pneumonia (median, 2.27x10^7^ cfu/ml), compared to children who survived (*p* = 0.003). These findings suggest that there might be a correlation between high nasopharyngeal density and disease severity, although further studies would be required to confirm these observations.

Nasopharyngeal bacterial density in children with respiratory infection (ARI, URTI, clinical pneumonia and pneumonia with normal CXR) was also found to be increased when evaluated for *Hi* and *Mc*, but not for *Sa* and *Nm*. While nasopharyngeal pneumococcal density has not been found useful to assist in the diagnosis of radiological pneumonia in children [8], our findings of a 2.5-fold, or 2-fold, increase in pneumococcal density in the nasopharynx of children with clinical pneumonia, or ARI, respectively, may facilitate secondary bacterial infection.

In healthy children the most common positive association seen in the nasopharynx is between *Sp* and *Hi*, whereas a negative association between *Sp* and *Sa* has been observed [31]. When we modeled our prevalence data, the strongest positive association was detected between *Sp* and *Mc*, followed by *Hi* and *Mc*, suggesting that *Mc* may drive the carriage of the other two species. Acquiring evidence to support whether Mc may play a central role in driving carriage of *Sp* and *Hi* will require further efforts. Carriage of *Mc* does not affect carriage of *Sa* or *Nm* as this bacterium, *Mc*, was neither negatively, nor positively associated with *Nm* or *Sa*, in contrast to both *Sp* and *Hi* which were both positively associated with *Nm* and negatively associated with carriage of *Sa*.

There are some limitations in this manuscript that need to be mentioned. For example, bacterial cultures were not obtained, and pneumococcal types were not investigated. The latter information may have been relevant in view that potential association between pneumococcal serotypes and individual species (i.e., *Mc*, *Hi*, *Sa*, or *Nm*) could not be explored. Another important limitation relates to the fact that viral infections have not been included in this paper, since the presentation of results would have been too complex.

In summary, our study demonstrated a significant association in febrile children between concurrent nasopharyngeal carriage of *Sp*, *Hi*, and *Mc* and respiratory infection. Firstly, when individually analyzed, carriage prevalence of *Sp*, *Hi*, or *Mc* was significantly increased in febrile ARI cases, cases of URTI, and children with clinical pneumonia. Secondly, when we considered all five species, our analyses showed that in the absence of *Sa* and *Nm*, concurrent carriage of *Sp*, *Hi*, and *Mc* was significantly more prevalent in the nasopharynx of children with clinical pneumonia and pneumonia with abnormal CXR, in comparison to non-ARI cases and URTI. Thirdly, when we assessed nasopharyngeal density, our study demonstrated a significantly increased density of *Sp*, *Hi*, and *Mc* in cases of febrile respiratory infection vs non-ARI. These findings call for the development of quantitative multiplex point-of-care tests, or at least semi-quantitative tests that would ideally allow better prediction of a LRTI.

## Supporting Information

S1 Table Concurrent nasopharyngeal carriage stratified by disease. (DOCX)

S2 Table Median bacterial density (cfu/ml) of each pathogen in the different respiratory diseases. (DOCX)
