# Supplementary material for: Increased Nasopharyngeal Density and Concurrent Carriage of Streptococcus pneumoniae, Haemophilus influenzae, and Moraxella catarrhalis Are Associated with Pneumonia in Febrile Children
Source: PLoS One. 2016 Dec 1;11(12):e0167725. doi: 10.1371/journal.pone.0167725 (PMC5132320; doi:10.1371/journal.pone.0167725)
Supplement: S1 Table — (DOCX) [file pone.0167725.s001.docx]

**S1 Table. Concurrent nasopharyngeal carriage stratified by disease.**

| Bacterial species # | Overall N=960 n (%) | Non-ARI N=363 n (%) | ARI N=597 n (%) | URTI N=420 n (%) | Clinical pneumonia N=177  n (%) | Pneumonia with normal CXR  N=117 n (%) | End-point Pneumonia N=60  n (%) |
| --- | --- | --- | --- | --- | --- | --- | --- |
| Sp | 8 (0.8) | 5 (1.4) | 3 (0.5) | 2 (0.5) | 1 (0.6) | 1 (0.8) | 0 (0.0) |
| Mc | 26 (2.7) | 11 (3.0) | 15 (2.5) | 10 (2.4) | 5 (2.8) | 5 (4.3) | 0 (0.0) |
| Hi | 6 (0.6) | 3 (0.8) | 3 (0.5) | 3 (0.7) | 0 (0.0) | 0 (0.0) | 0 (0.0) |
| Nm | 8 (0.8) | 4 (1.1) | 4 (0.7) | 3 (0.7) | 1 (0.6) | 0 (0.0) | 1 (1.7) |
| Sa | 8 (0.8) | 4 (1.1) | 4 (0.7) | 3 (0.7) | 1 (0.6) | 1 (0.8) | 0 (0.0) |
| Sp+Hi | 9 (0.9) | 3 (0.8) | 6 (1.0) | 6 (1.4) | 0 (0.0) | 0 (0.0) | 0 (0.0) |
| Sp+Nm | 4 (0.4) | 1 (0.3) | 3 (0.5) | 2 (0.5) | 1 (0.6) | 0 (0.0) | 1 (1.7) |
| Sp+Sa | 2 (0.2) | 1 (0.3) | 1 (0.2) | 0 (0.0) | 1 (0.6) | 1 (0.8) | 0 (0.0) |
| Sp+Mc | 53 (5.5) | 26 (7.2) | 27 (4.5) | 20 (4.8) | 7 (4.0) | 6 (5.1) | 1 (1.7) |
| Mc+Hi | 30 (3.1) | 11 (3.0) | 19 (3.2) | 13 (3.1) | 6 (3.4) | 5 (4.3) | 1 (1.7) |
| Mc+Sa | 7 (0.7) | 3 (0.8) | 4 (0.7) | 2 (0.5) | 2 (1.1) | 2 (1.7) | 0 (0.0) |
| Mc+Nm | 11 (1.1) | **8 (2.2)** | **3 (0.5)** | 3 (0.7) | 0 (0.0) | 0 (0.0) | 0 (0.0) |
| Hi+Nm | 4 (0.4) | 2 (0.6) | 2 (0.3) | 2 (0.5) | 0 (0.0) | 0 (0.0) | 0 (0.0) |
| Hi+Sa | 2 (0.2) | 0 (0.0) | 2 (0.3) | 2 (0.5) | 0 (0.0) | 0 (0.0) | 0 (0.0) |
| Nm+Sa | 6 (0.6) | **6 (1.7)** | **0 (0.0)** | 0 (0.0) | 0 (0.0) | 0 (0.0) | 0 (0.0) |
| Sp+ Hi+Mc | 223 (23.2) | **71 (19.6)*** | **152 (25.5)** | **94 (22.4)**** | **58 (32.8)** | **39 (33.3)^** | 19 (31.7) |
| Sp+Nm+Sa | 0 (0.0) | 0 (0.0) | 0 (0.0) | 0 (0.0) | 0 (0.0) | 0 (0.0) | 0 (0.0) |
| Sp+Nm+Mc | 43 (4.5) | 18 (5.0) | 25 (4.2) | 18 (4.3) | 7 (4.0) | 4 (3.4) | 3 (5.0) |
| Sp+Sa+Mc | 16 (1.7) | 8 (2.2) | 8 (1.3) | 7 (1.7) | 1 (0.6) | 1 (0.8) | 0 (0.0) |
| Sp+Nm+Hi | 6 (0.6) | 4 (1.1) | 2 (0.3) | 2 (0.5) | 0 (0.0) | 0 (0.0) | 0 (0.0) |
| Mc+Hi+Nm | 20 (2.1) | 7 (1.9) | 13 (2.2) | 9 (2.1) | 4 (2.3) | 3 (2.6) | 1 (1.7) |
| Mc+Hi+Sa | 7 (0.7) | 3 (0.8) | 4 (0.7) | 4 (1.0) | 0 (0.0) | 0 (0.0) | 0 (0.0) |
| Mc+Nm+Sa | 9 (0.9) | 6 (1.7) | 3 (0.5) | 3 (0.7) | 0 (0.0) | 0 (0.0) | 0 (0.0) |
| Hi+Sa+Nm | 1 (0.1) | 0 (0.0) | 1 (0.2) | 1 (0.2) | 0 (0.0) | 0 (0.0) | 0 (0.0) |
| Sp+ Hi+Sa+ Mc | 47 (4.9) | 14 (3.9) | 33 (5.5) | 22 (5.2) | 11 (6.2) | 6 (5.1) | 5 (8.3) |
| Sp+ Nm+ Sa+Mc | 20 (2.1) | 10 (2.8) | 10 (1.7) | 10 (2.4) | 0 (0.0) | 0 (0.0) | 0 (0.0) |
| Sp+Nm+Mc+Hi | 262 (27.3) | **84 (23.1)** | **178 (29.8)** | 128 (30.5) | 50 (28.3) | 29 (24.8) | 21 (35.0) |
| Mc+Hi+Nm+Sa | 15 (1.6) | **12 (3.3)** | **3 (0.5)** | 2 (0.5) | 1 (0.6) | 0 (0.0) | 1 (1.7) |
| Sp+ Hi+Nm+Sa+Mc | 83 (8.6) | 28 (7.7) | 55 (9.2) | 37 (8.8) | 18 (10.2) | 14 (12.0) | 4 (6.7) |
| No organism | 24 (2.5) | 10 (2.8) | 14 (2.3) | 12 (2.9) | 2 (1.1) | 0 (0.0) | 2 (3.3) |

Note: those in bold are the ones with significant *p* values by two-by-two tables.

* P-value (2-tail) 0.03514, OR 1.40 95% CI (1.024 -1.937) Non-ARI vs ARI

** P-value (2-tail) 0.008947, OR 1.689 95% CI (1.142 - 2.491) URTI vs clinical pneumonia

^ P-value (2-tail) 0.01801, OR 1.734, 95% CI (1.108 – 2.713) URTI vs pneumonia with normal CXR

# The absence of bacterial species in each row should be considered negative. e.g., Sp=Sp+, Mc-, Hi-, Nm- and Sa- .
