# Supplementary material for: Increased Nasopharyngeal Density and Concurrent Carriage of Streptococcus pneumoniae, Haemophilus influenzae, and Moraxella catarrhalis Are Associated with Pneumonia in Febrile Children
Source: PLoS One. 2016 Dec 1;11(12):e0167725. doi: 10.1371/journal.pone.0167725 (PMC5132320; doi:10.1371/journal.pone.0167725)
Supplement: S2 Table — (DOCX) [file pone.0167725.s002.docx]

**S2 Table. Median bacterial density (cfu/ml) of each pathogen in the different respiratory diseases.**

|  | **Febrile disease category** | | | | | | | |
| --- | --- | --- | --- | --- | --- | --- | --- | --- |
|  |  |  |  |  | **Pneumonia with:** | |  | |
| **Species** | **non-ARI** | **ARI** | **URTI** | **Clinical pneumonia** | **Normal CXR** | **abnormal CXR** | **Severe pneumonia** | **mild URTI**** |
| ***Sp*** | 9.2x10^5^ | 1.9x10^6^ | 1.7x10^6^ | 2.0x10^6^ | 2.3x10^6^ | 1.8x10^6^ | 7.0x10^6^ | 1.7x10^6^ |
| p-value* | NA | <0.001 | 0.004 | <0.001 | <0.001 | 0.045 | NA | <0.002 |
| ***Hi*** | 1.8x10^6^ | 4.9x10^6^ | 3.6x10^6^ | 7.6x10^6^ | 7.6x10^6^ | 7.6x10^6^ | 2.5x10^7^ | 3.7x10^6^ |
| p-value* | NA | <0.001 | 0.003 | <0.001 | <0.001 | 0.004 | NA | <0.001 |
| ***Mc*** | 3.5x10^6^ | 6.1x10^6^ | 5.1x10^6^ | 7.7x10^6^ | 7.8x10^6^ | 6.7x10^6^ | 1.2x10^7^ | 5.1x10^6^ |
| p-value* | NA | <0.001 | <0.001 | <0.001 | <0.001 | 0.005 | NA | 0.014 |
| ***Sa*** | 3.5x10^4^ | 2.0x10^4^ | 2.5x10^4^ | 1.2x10^4^ | 1.2x10^4^ | 3.2x10^4^ | 2.3x10^3^ | 2.6x10^4^ |
| p-value* | NA | 0.249 | 0.455 | 0.156 | 0.050 | 0.723 | NA | 0.191 |
| ***Nm*** | 3.9x10^4^ | 2.4x10^4^ | 2.2x10^4^ | 2.8x10^4^ | 2.9x10^4^ | 2.8x10^4^ | 3.3x10^4^ | 2.3x10^4^ |
| p-value* | NA | 0.092 | 0.090 | 0.348 | 0.477 | 0.462 | NA | 0.212 |

*Nasopharyngeal density of the indicated category was compared against non-ARI cases.

** Nasopharyngeal density of children with mild URTI was compared against those with severe pneumonia.
